# Analysis of communication styles underpinning clinical decision-making in cancer multidisciplinary team meetings

**DOI:** 10.3389/fpsyg.2023.1105235

**Published:** 2023-05-02

**Authors:** Tayana Soukup, Benjamin W. Lamb, James S. A. Green, Nick Sevdalis, Ged Murtagh

**Affiliations:** ^1^Health Service and Population Research Department, Centre for Implementation Science, King's College London, London, United Kingdom; ^2^Department of Surgery and Cancer, Imperial College London, London, United Kingdom; ^3^Department of Epidemiology and Environmental Health, University of Buffalo, Buffalo, NY, United States; ^4^School of Health and Sport Sciences, University of Suffolk, Suffolk, United Kingdom; ^5^Whipps Cross University Hospital, Barts Health NHS Trust, London, United Kingdom; ^6^Barts Cancer Institute, Queen Mary University of London, London, United Kingdom

**Keywords:** cancer multidisciplinary teams, multidisciplinary tumor boards, teamwork, communication, interaction, teamwork among the medical professions, clinical decision-making, multidisciplinary team meetings

## Abstract

**Introduction:**

In cancer care, multidisciplinary team (MDT) meetings are the gold standard. While they are trying to maximize productivity on the back of the steadily increasing workload, growing cancer incidence, financial constraints, and staff shortages, concerns have been raised with regards to the quality of team output, as reported by Cancer Research UK in 2017: “*Sometimes we discuss up to 70 patients. This is after a whole day of clinics, and we do not finish until after 19.00. Would you want to be number 70?*”. This study aimed to explore systematically some of the dynamics of group interaction and teamwork in MDT meetings.

**Materials and methods:**

This was a prospective observational study conducted across three MDTs/university hospitals in the United Kingdom. We video-recorded 30 weekly meetings where 822 patient cases were reviewed. A cross-section of the recordings was transcribed using the Jefferson notation system and analyzed using frequency counts (quantitative) and some principles of conversation analysis (qualitative).

**Results:**

We found that, across teams, surgeons were the most frequent initiators and responders of interactional sequences, speaking on average 47% of the time during case discussions. Cancer nurse specialists and coordinators were the least frequent initiators, with the former speaking 4% of the time and the latter speaking 1% of the time. We also found that the meetings had high levels of interactivity, with an initiator–responder ratio of 1:1.63, meaning that for every sequence of interactions initiated, the initiator received more than a single response. Lastly, we found that verbal dysfluencies (laughter, interruptions, and incomplete sentences) were more common in the second half of meetings, where a 45% increase in their frequency was observed.

**Discussion:**

Our findings highlight the importance of teamwork in planning MDT meetings, particularly with regard to Cancer Research UK in 2017 cognitive load/fatigue and decision-making, the hierarchy of clinical expertise, and the increased integration of patients' psychosocial information into MDT discussion and their perspectives. Utilizing a micro-level methodology, we highlight identifiable patterns of interaction among participants in MDT meetings and how these can be used to inform the optimization of teamwork.

## 1. Introduction

In the United Kingdom (UK), multidisciplinary teams (MDTs or tumor boards) routinely plan care management for people with cancer. This generally consists of histopathologists, radiologists, surgeons, specialist cancer nurses (CNSs), and oncologists. They typically meet weekly or bi-weekly, and they discuss large numbers of cancer cases for several hours at a time (Department of Health, 2004; Raine et al., 2014; Cancer Research UK, 2017; Soukup et al., 2018, 2019a; National Institute for Health Care Excellence, 2020; Guirado et al., 2022).

**Figure 1:**
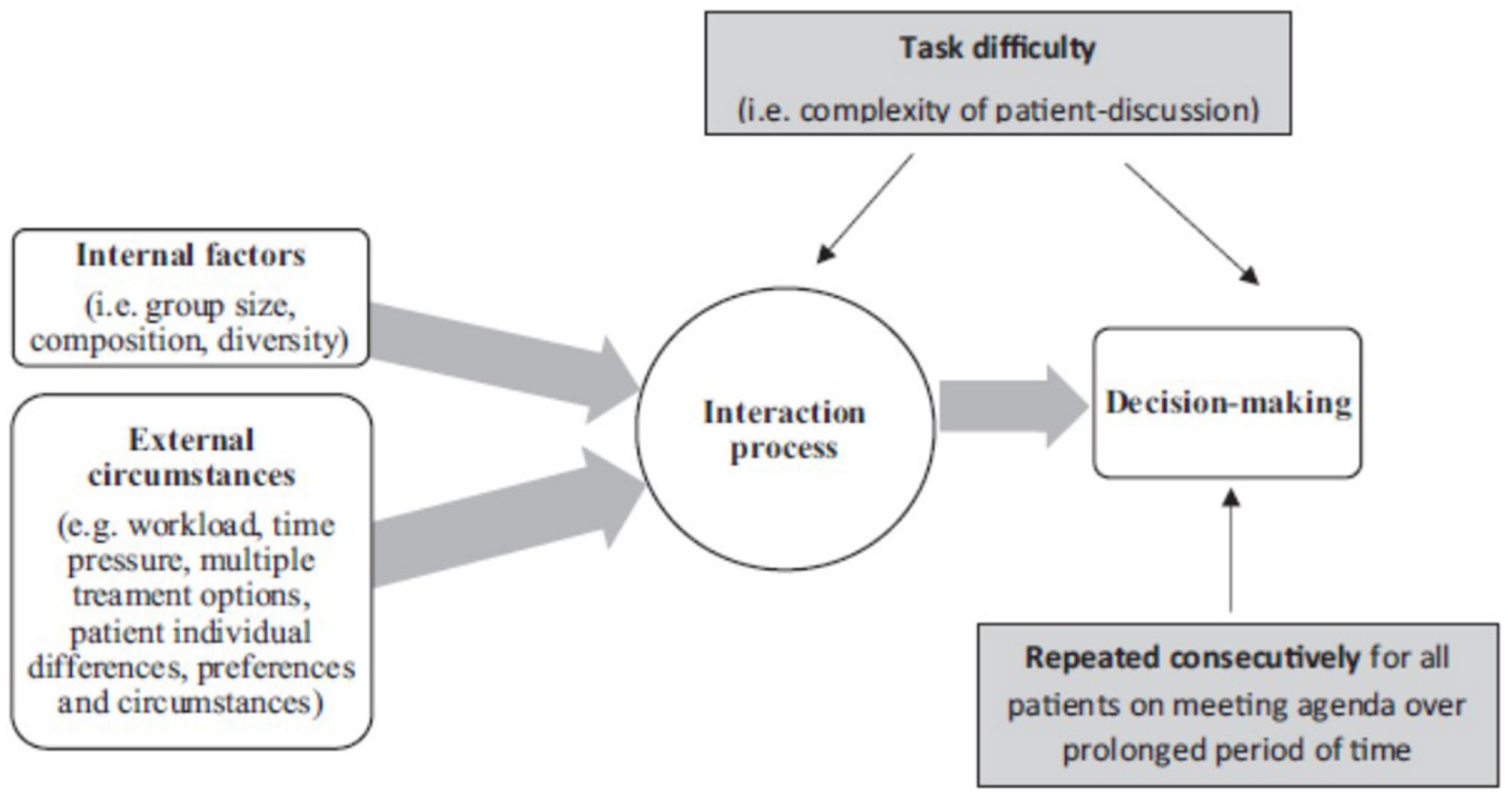
Graphic representation of the factors affecting cancer team functioning and communication in line with the functional perspective of group decision-making. This diagram demonstrates the functional perspective of group decision-making graphically with the interaction process and communication at the center of the process. Reprinted with permission from the following two sources: (Soukup, 2017) “Socio-cognitive factors that affect decision-making in cancer multidisciplinary team meetings [Doctoral thesis, Imperial College London]” Spiral Repository, https://doi.org/10.25560/79603 by Soukup (2017); and (Soukup et al., 2020a) “A multicenter observational cross-sectional observational study of cancer multi- disciplinary teams: analysis of team decision-making” by Soukup et al. (2021c), Cancer Medicine, https://doi.org/10.1002/cam4.3366.CCBY-NC-ND.

While the MDT model is considered the gold standard (Raine et al., 2014; National Institute for Health Care Excellence, 2020), evidence indicates that MDTs are often subject to a variety of internal and external factors that may influence their functioning and, more specifically, the communication process between the team members (Lamb et al., 2011, 2013; Raine et al., 2014; Soukup et al., 2016a,b, 2020a,b, 2021c). For example, factors external to the team (see Figure 1) may include things such as time and workload pressures. A recent large-scale study into MDT communication and decision-making (Soukup et al., 2020a) found a reduction in the frequency of task-oriented communication (e.g., asking questions and giving answers to those questions) in the second half of meetings, possibly because of the experience of fatigue later in the meeting. This is in addition to such effects found with the quality of decision-making with cases discussed at the beginning of meetings generally receiving more discussion (Lamb et al., 2013; Soukup et al., 2019a,b, 2020a). As cancer MDTs try to maximize productivity in the face of ever-increasing workload (Cancer Research UK, 2017), growing cancer incidence (NHS England, 2014; World Health Organization, 2014), and complexities around repeated recurrence of cancer, for which treatment options are not necessarily standardized by the (inter)national guidelines (in contrast to treatment options for first occurrence), in addition to financial constraints (Mistry et al., 2011; NHS England, 2014), and the pressures brought by staff shortages (NHS Improvement, 2016), concerns have been raised that the quantity of workload of MDT meetings negatively impacts on the quality of output (Cancer Research UK, 2017). In the Cancer Research report published in 2017 (Cancer Research UK, 2017), one MDT member was quoted as saying: “*Sometimes we discuss up to 70 patients. This is after a whole day of clinics, and we do not finish until after 19.00. Would you want to be number 70?*” (Cancer Research UK, 2017).

Factors internal to the team (Figure 1) may involve further possible impediments to team communication. For example, MDT meetings can be fast-paced, particularly for the uninitiated. This means that securing one's turn to contribute may be challenging, potentially reducing levels of participation by team members (Soukup et al., 2021c), leading to unequal contributions and suboptimal sharing of information (Lamb et al., 2013; Raine et al., 2014; Soukup et al., 2020a,b, 2021c). While the reasons behind the underutilization of expertise in meetings are not fully understood (Valcea et al., 2019), the significance of it cannot be overlooked. For instance, a recent study of MDT meetings (Soukup et al., 2020a) found that higher levels of interactive responsiveness among team members significantly predicted better quality decision-making for patients. Indeed, communication is the channel through which the team progresses through the stages of decision-making: from problem identification, information sharing, and critical evaluation to formulating the decision and implementing it (Orlitzky and Hirokawa, 2001; Hollingshead et al., 2005; Kugler et al., 2012; Soukup, 2017; Soukup et al., 2020b, 2021a). There is then a need to build an understanding of the communication practices team members employ during their meetings and how this can be improved.

This study aimed to systematically explore some of the dynamics of group interaction in MDT meetings. To do this, the study employed a linguistic analysis previously used in cancer MDTs (Soukup et al., 2021a,c), which includes a combination of quantitative frequency counts, and a qualitative approach based on the principles of conversation analysis (CA), which details characteristics of speech exchange (e.g., questions and answers, pauses, pace, and intonation; 25–26). We used this forensic approach for the analysis of speech and interaction to gain an understanding of how decision-making is shaped interactionally and how the levels of participation are shaped during case discussions. More specifically, we attempted to address the following issues:

Q1: Is there an identifiable pattern for who leads or initiates talk in these meetings?
Q2: How responsive are team members to one another during case discussions?
Q3: Is there a difference in communication in the first vs. the second half of the meeting?

## 2. Materials and methods

### 2.1. Study design

This was a prospective, cross-sectional, observational study.

### 2.2. Study setting

We recruited three cancer MDTs (breast, colorectal, and gynecologic) from three university hospitals in the Greater London and Derbyshire areas of the UK. Their meetings were video recorded for 3 months each. The study took place between September 2015 and July 2016. The study was granted ethical and regulatory approvals from the Northwest London Research Ethics Committee (JRCO REF. 157441) and the R&D departments of the participating NHS Trusts. Informed consent was obtained from the MDT members. Patient consent was not required because patient-identifiable information was retained during the study. This study was part of a larger MDT study (Soukup, 2017) that adopted by the the National Institute for Health Research Clinical Research Network Portfolio.

### 2.3. Participants and sample size

Availability sampling was used to identify the MDTs. The criterion for the study was a cancer MDT from the UK National Health Service (NHS) that represents the most common type of cancer. The recruited participants were 44 MDT members from across three cancers: breast, colorectal, and gynecologic. The teams consisted of surgeons, oncologists, CNSs, radiologists, histopathologists, and coordinators (medical students sometimes attended on an educational basis). At least one team member from each professional group was present during the MDT meetings, with an average attendance presented in Table 1. However, all participating MDTs organized their cases on the meeting agenda in line with whether the case required radiologists' input only, histopathologists' input only, or both radiologists' and histopathologists' inputs—this influenced at what point during the meeting the radiologists and histopathologists came into the room. Further details on team composition and meeting characteristics are found in Table 1.

**Table 1:** Team composition and meeting characteristics of participating cancer multidisciplinary teams.

**Variable**	**Cancer multidisciplinary team**							
	**Breast**		**Colorectal**		**Gynecologic**		**Full sample**	
	***N***	***n*** **of women**	***N***	***n*** **of women**	***N***	***n*** **of women**	***N***	***n*** **of women**
**Team composition**								
Surgeons	4	2	4	1	4	0	12	3
Oncologists	2	2	2	1	2	2	6	5
Radiologists	2	1	2	0	2	2	6	3
Pathologists	1	1	1	0	3	2	5	3
Specialist cancer nurses	5	5	5	4	2	2	12	12
Team coordinator	1	1	1	1	1	1	3	3
Total	15	12	15	7	14	10	44	30
**Meeting characteristics**								
Average number of members present	11	7	11	6	7	2	9	5
Average number of cases discussed per meeting	26		20		43		33	
Average time per patient (HH:MM:SS)	00:02:25		00:03:20		00:02:30		00:02:58	
Average meeting duration (HH:MM:SS)	01:06:00		01:00:00		02:52:00		01:53:00	
**Study characteristics**								
Number of hours recorded (HH:MM:SS)	09:57:00		13:40:00		31:30:00		55:07:00	
Number of cases discussed	241		185		396		822	
Number of meetings observed	10		10		10		30	

*n* = subsample size of female members within each cancer multidisciplinary team. *N* = sample size within each team. Reprinted with permission from the following two sources: (Soukup, 2017) “Socio-cognitive factors that affect decision-making in cancer multidisciplinary team meetings [Doctoral thesis, Imperial College London].” Spiral Repository, https://doi.org/10.25560/79603 by Soukup, 2017; and (Soukup et al., 2021c) “Gaps and overlaps in multidisciplinary team meetings: analysis of speech” by Soukup et al. (2021c), Small Groups Research, https://doi.org/10.1177/1046496420948498.CC~BY-NC-ND.

A total of 822 case discussions were video recorded. These consisted of all the cases listed on the meeting agenda (including suspected or confirmed cancers and, in breast and gynecologic cancer teams, benign cases) discussed in 30 meetings (or 55 h of meeting time). A selection of 24 malignant discussions is presented in this article (or 72 min of meeting footage). The selection criteria for the 24 case discussions have been described in some detail previously (Soukup, 2017; Soukup et al., 2021a,c), and they included the following: quality and clarity of the audio, feasibility, equal distribution between the first and second half of the meetings, duration of the case discussion, malignancy, and saturation (Soukup, 2017; Soukup et al., 2021a,c).

The long-term approach that we use in filming MDT meetings is something that we have described in some detail previously (Soukup, 2017; Soukup et al., 2021a,c). Such an approach entails the following: (a) filming the team for at least 3 months (12 consecutive weekly meetings); (b) excluding the first two meetings from analysis as these were used to allow the team to get used to being observed/filmed and for the assessor to learn about who is who in the team (for example, although we collected the data over 30 meetings, we recorded 36 meetings, allowing us to exclude six meetings or the first two meetings for each team); (c) filming was conducted discreetly with a small camera (all sound off, operated remotely) and out of the immediate view of the team (placed with other meeting room equipment). Such strategies help to induce habituation, allowing the teams to “forget” about the camera and continue their practice as usual, therefore, minimizing the Hawthorne effect (Soukup, 2017; Soukup et al., 2021a,c).

The layout of the meeting rooms each MDT used did not change during the study. Each room had two large screens: one for patient proforma and the other for radiology/histopathology slides. All attendees were seated in a U-shape facing the large screens, making the behavior of all attendees straightforward to capture with a single camera. The breast and gynecologic MDT meetings were conducted in a face-to-face format, with all core disciplines physically present during case discussions. This was in contrast to the colorectal MDT meetings, which were hybrid, with the histopathologist and oncologist having to dial into the meeting virtually from another hospital site.

### 2.4. Materials

We examined communication in the MDT meetings by capturing not only what was said but also how it was said. We used the Jefferson notation system, commonly used in CA (Psathas, 1994; Ten Haves, 2007), to identify and analyze different aspects of communication and interaction during case discussions. We combined qualitative and quantitative approaches in our analyses. While the former is traditionally used in the CA, the latter approach uses frequency counts and has been used on the individual case discussions in previous research utilizing CA (Stivers, 2001, 2002; Soukup, 2017; Soukup et al., 2021a,c), and more frequently in linguistics (Ten Bosch et al., 2004; Kurtić et al., 2013; Levinson and Torreira, 2015).

For quality control and as a vital part of CA (Ten Haves, 2007), our data have been discussed in multiple data sessions (*N* = 4) with leading international CA scholars, who provided their critical input and insight into the analysis presented in this study. This included watching videos of MDT meetings and discussing the interaction while formulating points of interest in the data and how best to analyze such complex multiparty interactions.

### 2.5. Analyses

#### 2.5.1. Q1: Is there an identifiable pattern of who leads or initiates talk in the meetings?

Here, we aimed to determine several things using CA. First, how the interaction was initiated in these meetings; second, whether some groups initiate interaction more frequently than others; and lastly, levels of responsiveness, i.e., did some groups respond more often than others, and how did they respond? We have identified grammatical constructs (21, 25–26, 32–33; shown in Table 2), which we grouped against individual disciplines comprising an MDT (i.e., surgery, radiology, histopathology, nursing, and oncology). For each discipline and team, the usage frequency of these actions was calculated using counts and percentages.

**Table 2:** Overview of terms used in the analysis of communication among participating cancer multidisciplinary teams (MDTs).

**Discourse and dimension**	**Example quote**
**Declarative form**	
•1.a Giving information to others.	**PAT:** “It is an invasive high-grade serous adenocarcinoma.”
**Interrogative form**	
•2.a Seeking information from others.	**ONC**: “Has she got some other malignancy going on?”
**Imperative form**	
•3.a Giving instructions to others.	**ONC:** “Write it on the MDT outcome sheet.”
**Adjacency pair**	
•4.a A basic unit of interaction that is typically paired, e.g., a question is typically followed by an answer	[e.g. question-answer pair]
	**ONC: “**Has she got some other malignancy going on?”
	**RAD: “**Well, there is something in the lung.”
	[e.g. request-compliance]
	**ONC**: “Write it on the MDT outcome sheet.”
	**NUR:** “Okay.”
**Originator/initiator**	
•5.a The person that initiates the interactional sequence.	**ONC: “**Has she got some other malignancy going on?”
**Responder**	
•6.a The person that responds to the originator's interactional sequence.	**RAD: “**Well, there is something in the lung.”

SUR, surgeon; PAT, pathologist; NUR, nurse; ONC, oncologist; RAD, radiologist; MDT, multidisciplinary team. Reprinted with permission from Soukup (2017) “Socio-cognitive factors that affect decision-making in cancer multidisciplinary team meetings [Doctoral thesis, Imperial College London].” Spiral Repository, https://doi.org/10.25560/79603 by Soukup (2017).

#### 2.5.2. Q2: How responsive are team members to one another during case discussions?

We calculated the degree of responsiveness (to the initiator's utterance, question, or request) during case discussions using the originator–responder ratio (Soukup, 2017). Here, the total number of responses was divided by the total number of sequences prompted by the initiator of the interaction (Soukup, 2017).

#### 2.5.3. Q3: Is there a difference in communication in the first vs. the second half of the meeting?

Here, we explored cognitive load as linguistically evident through verbal fragmentations and dysfluencies, such as incomplete sentences and interruptions, pauses, pitch peaks, repetitions, vocalizations, interruptions, laughter, and chatter (Bortfeld et al., 2001; Arnold et al., 2003; Adda-Decker et al., 2008; Corley and Stewart, 2008; Soukup, 2017). An association between high levels of such verbal behaviors and higher levels of cognitive load and fatigue was previously found (Arnold et al., 2003; Heldner and Edlund, 2010; Nicholson et al., 2010; Womack et al., 2012). In addition, we determined the frequency of the identified verbal fragmentations in the transcripts across the first and second halves of the meetings. Table 3 shows a list of fragmentations with the corresponding definitions, symbols, and data examples that were examined across all three MDTs. Frequencies were converted to a percentage change from the first to the second half of the meetings.

**Table 3:** List of verbal fragmentations, and corresponding definitions, Jefferson notation symbols, and data examples.

**Discourse and dimensions**	**Example quotes**
**Incomplete sentence**	**ON: so I am not/** and I think we need to review everything for this lady.
•A sentence, phrase, or word that is too incomplete to be understood.	—————–
•A forward slash (**/**).	**ON: Could/** does it say why?
**Interruption (overlaps and cut-offs)**	**PAT:** no, the only**[**thing is the-**]**
•**“**A successful speaker switch in which there is some simultaneous talk, but the first speaker's utterance is not completed and the incoming speaker has successfully gained the floor” (Hutchby and Wooffitt, 2008, p. 110). Cooperative recognition of the first speaker's overlapping point was not counted.	**ONC: [**so you just**]** have a chest x-ray?
•Overlap is indicated by square brackets **[]**, and cut off by a dash (**-**).	——————— **RAD:** so **[**this is-**] SUR: [**they have**]** all been interesting today, every single one of them
**Laughter and chatter (break in communication flow)**	**SUR:** she was worried hmm::
•Temporary break in communication flow (normally related to the formulation of a treatment plan) that needs to then be reestablished later. Includes laughter and chatter about an unrelated topic.	**((laughter from many in the room))**
•Double brackets.	**ONC:** yes but I can not believe there were five appointments
**Pauses**	**ONC:** ye::s but I can not believe there were five **(0.4)** appointments that she was **(0.4)** DNA as a result
•Continuous pause segment of more than 100 milliseconds/ 0.1 seconds between words, or sentences was counted.	————–
•Number in brackets.	**NUR:** someone needs to call **(2.4)**
**Pitch peaks**	ONC:**so** **↑you** **↑just** **↑have a** **↑chest x-ray?**
•Shifts to a particularly high-pitch, or loud speech relative to the surrounding speech.	—————
•Up-facing arrow (**↑**), upper case.	SUR:**uh I PRESUME YOU DO NOT HAVE ANY HISTOLOGY, ↑right?**
**Repetitions**	**PAT: we/ we** looked at it
•Repetition of words or groups of words incorporated in a sentence.	————–
•Repetition.	**NUR: shall we/ shall we** look at
**Vocalizations**	**ONC:** a::nd **um** only had radiotherapy at that poi:nt as was appropriate **um** and the::n, she was followed up for a number of years, but **um**
•In the struggle to find a word, the speaker is compelled to insert a sound to repair the break in the flow of communication (also known as vocal insertions).	——————
•ah, eh, er, aw, uh, um, hm, mm	**SUR:** this is the chap that had an adenocarcinoma **er**

m, man; f, woman; SUR, surgeon; PAT, pathologist; NUR, nurse; ONC, oncologist; RAD, radiologist. Reprinted with permission from Soukup (2017) “Socio-cognitive factors that affect decision-making in cancer multidisciplinary team meetings [Doctoral thesis, Imperial College London].” Spiral Repository, https://doi.org/10.25560/79603 by Soukup (2017).

## 3. Results

### 3.1. Q1: Is there an identifiable pattern of who leads or initiates conversation in the meeting?

Table 4 shows that the higher levels of verbal contribution in breast cancer MDT meetings were made by surgeons and oncologists. These two professional groups were also frequent initiators, i.e., they typically started the discussion and answered the questions about the case (e.g., Case 16, Surgeon: “This is a 26 year-old presenting with intermittent spontaneous discharge.” and Case 12, Oncologist: “We will need to keep an eye out for HER2.”). The most frequent initial questions come from the oncologists (e.g., Case 2, Oncologist: “Why did she start on Letrizole?”; Case 12, Surgeon: “Has she had a CT?”). Radiologists were also frequent contributors to the discussion together with, but to a lesser extent, pathologists (e.g., Case 10, Oncologist: “What was the biopsy result?”, Pathologist: “It was benign.”; Case 8, Surgeon: “Are you happy [with the images], Mark [the radiologist]?”, Radiologist: “Yeah”, Surgeon: “Yeah fine okay … R&D”).

**Table 4:** Communication style by professional group across participating cancer multidisciplinary teams (MDTs).

**Professional group**	***n***	**SPEAKING %**	**Originator %**	**Responder %**	**Originator**			**Responder**		
					**Declarative %**	**Interrogative %**	**Imperative %**	**Declarative %**	**Interrogative %**	**Imperative %**
**Breast cancer MDT**										
Surgeon	4	39	11	28	14	11	3	37	4	4
Oncologist	2	28	16	12	23	17	3	17	0.4	2
Radiologist	2	16	2	14	4	2	–	20	0.4	0.4
Pathologist	1	13	8	5	13	4	3	8	0.4	0.4
Cancer nurse specialist	5	4	1	3	–	3	–	6	–	–
Coordinator	1	0.3	–	0.3	–	–	–	0.4	–	–
Overall	15	100	38	62	54	37	9	88	5	6
**Colorectal cancer MDT**										
Surgeon	4	63	43	20	32	43	15	35	–	4
Oncologist	2	1	–	1	–	–	–	1	–	–
Radiologist	2	15	2	13	1	1	–	26	–	–
Pathologist	1	5	–	5	–	–	–	10	–	–
Cancer nurse specialist	5	15	3	12	2	6	–	22	–	–
Coordinator	1	1	–	1	–	–	–	2	–	–
Overall	15	100	48	52	35	50	15	94		4
**Gynecologic cancer MDT**										
Surgeon	4	40	30	10	41	10	5	20	–	–
Oncologist	2	14	6	8	9	2	2	16	–	–
Pathologist	2	21	11	10	18	–	1	22	1	–
Radiologist	3	14	3	11	5	–	1	23	1	–
Cancer nurse specialist	2	10	3	7	6	–	–	12	3	–
Coordinator	1	1	–	1	–	–	–	2	–	–
Overall	14	100	53	47	79	12	9	95	5	–

*N*, 24 case discussions; MDT, multidisciplinary team. The originator–responder ratio in the gynecologic cancer team it was 1:1.13, in the breast cancer team it was 1:1.63, and in the colorectal cancer team it was 1:1.1.

In contrast, the least frequent speakers in the breast cancer MDT meetings were CNSs and coordinators. Their contributions typically took the form of a response to something the surgeon had raised, “Those scans?”, i.e., providing information and facts (e.g., Case 12, Oncologist: “Has she had a lung MDT discussion?”, Nurse: “No”; Oncologist: “Can you get them?”, Coordinator: “Those scans” Oncologist: “Yes, please yeah”). However, the data showed that the CNSs sometimes initiated an interaction (for example in Case 8, “Does anyone want to see the abscess?”) which appeared to lead to a change from the original decision of “Reassure and discharge” to “Clinical review”.

In the colorectal cancer MDT meetings, the surgeons were also the most frequent contributors to the meetings. They typically used questions to initiate interaction (e.g., Case 4, “Do you you have this, Paul [pathologist]?”, “Okay, is it suspicious for cancer?”; Case 11, “Did you see anything on the PET?”), but also declarative statements (e.g., Case 13, “This is his first request”). They were the only professional group to request actions (e.g., Case 4, “Will you document a reasonable request?”; Case 11, “For UA and excision”; Case 12, “So first refer to HPB for discussion, secondly refer to Dr. Sheppard to consider palliative chemotherapy”). In the colorectal cancer MDT meeting, those who most frequently responded to contributions by the surgeon were the radiologists and CNSs, who used largely declarative statements to provide information (e.g., Case 11, Surgeon: “Did you see anything on the PET?” Radiologist: “Well, there are two things...”; Case 14, Surgeon: “We do not need to do a colonoscopy, do we?” Nurse: “It is already booked.”), and to a lesser extent, they asked questions (e.g., Case 15, Nurse: “So who is going to follow her up?”; Case 14, Radiologist: “Did she have a colonoscopy?”). In these meetings, pathologists and coordinators contributed the least. When they did contribute, it was largely in response to a question or request from the surgeon. For example, in Case 12, Surgeon: “Do you have any histology report?”, Pathologist: “Very necrotic cause… which would be consistent with a colorectal primary”; or Case 3, Surgeon: “Hold on a second, Anna [the coordinator] is checking?”, Coordinator: “We have him scheduled for the 24th”.

In the gynecologic cancer MDT meetings, once again, surgeons were the ones who contributed the most, followed by histopathologists and, to a lesser extent, radiologists, oncologists, and CNSs. Surgeons spoke the most, using predominantly declarative statements to initiate interaction (e.g., Surgeon: “This is a lady who probably had stage 3 ovarian cancer, she has had an ultrasound-guided biopsy.” Pathologist: “Yeah, it is an invasive high-grade.”), but also interrogative (e.g., Case 27, “Is that the fairly simple cyst?”), and imperative (e.g., Case 27, “for THO and BSN”; Case 1, “So, discuss surgery vs. chemo”). In the gynecologic cancer MDT meetings, coordinators were also the least frequent speakers, responding largely in a declarative form (e.g., Case 37, Surgeon: “What is her CA 125?”, Coordinator: “123”).

### 3.2. Q2: How responsive are team members to each other during case discussions?

Breast cancer MDT members appeared highly responsive, with an initiator–responder ratio of 1:1.63, i.e., for every initiated sequence of interaction, the initiator received more than a single response. Colorectal and gynecologic cancer MDTs were also relatively responsive, with an initiator–responder ratio of 1:1.11 and 1:1.13, respectively, i.e., for every initiated sequence of interaction, the originator received a single response.

#### 3.2.1. Similarities between cancer teams

The coordinators' contributions appeared to be minimal at 1%, and they were in a declarative form, i.e., giving information. Across the participating teams, the CNSs did not appear to be making requests. Instead, the CNSs' inputs to the discussion were in the form of statements and questions, typically in response to others. One notable contribution (mentioned earlier) from the CNSs led to an amendment to the original recommendation for the patient. In this particular case, the patient is reported (by the pathologist and radiologist) to have a benign abscess. Asking for the team's opinion, the surgeon is met with a question from the CNS, which leads to a 3-min discussion and then the decision to review the case (line 73).

**12 Surgeon 2:** Are you happy?
**13 Radiologist:** Yeah.
**14 Surgeon 1:** Yeah, fine, okay.
**15 Surgeon 3:** Mh.
**16 Surgeon 2:** R&D?
**17 Surgeon 1:** Yeah.
**18 Nurse:** Does anyone want to see the abscess?

[3-min long exchange surgeons, radiologist, pathologist, and nurse regarding a plan of care]

**72 Surgeon 2:** Why not do a review?
**73 Surgeon 3:** Clinical review.

The teams also had in common the discipline that tended to formulate treatment recommendations for patients, which were most frequently surgeon-led and to a lesser extent oncologist-led. Moreover, another similarity across the participating teams was that the new information/knowledge about the patient and their circumstances were brought into the discussion by a wider range of disciplines, including surgeons, radiologists, pathologists, and to a lesser extent oncologists and CNSs. The type of information/knowledge that each discipline brought to the discussion corresponded to their area of expertise and how well they knew the patient. For instance:

**Clinical picture**

**Surgeon 3:** This is an 89-year-old woman who presented with a large mass in her right breast, graded T4, who had a mammogram and an ultrasound scan, and a core biopsy.

**Pathologist:** Okay, so she has an invasive ductal grade 2 carcinoma ER+ PR+ malignant invasive.

**Radiologist:** Yeah, in terms of imaging, it looks as if she has a primary… lesion in the cecum.

**Oncologist:** I brought her in, she is on adjuvant chemotherapy for stage 1 submucous cancer this year.

**Wider patient context**

**Nurse:** You have no follow-up.

**Nurse:** They [the patient and their family] are not happy about the wait, and they want to go and see Mr. Brown.

Table 5 summarizes the similarities and differences in multidisciplinary communication between the participating teams.

**Table 5:** Overview of similarities and differences in communication among participating cancer multidisciplinary teams (MDTs).

**Variable**	**Cancer multidisciplinary team**		
	**Breast**	**Colorectal**	**Gynecologic**
Most frequent speaker	**Surgeons**, oncologists	**Surgeons**	**Surgeons**, pathologists, radiologists, oncologists, CNSs
Least frequent speaker	**Coordinator**, CNSs	**Coordinator**, pathologists, oncologists	**Coordinator**
Most frequent originator	**Surgeons**, oncologists, pathologists	**Surgeons**	**Surgeons**, pathologists
Least frequent originator	**Coordinator**, radiologists, CNSs	**Coordinator**, oncologist, pathologist	**Coordinator**, oncologists, radiologists, CNSs
Most frequent responder	**Surgeons, radiologists**, oncologists	**Surgeons, radiologists**, CNSs	**Surgeons, radiologists**, pathologists, oncologists, CNSs
Least frequent responder	**Coordinator**, pathologists, CNSs	**Coordinator**, pathologists, oncologists	**Coordinator**
Originator-responder ratio	1:1.63	1:1.1	1:1.13
Common communication style	Declarative	Interrogative	Declarative

CNS, cancer nurse specialist. Similarities between teams are shown in bold. Reprinted with permission from Soukup (2017) “Socio-cognitive factors that affect decision-making in cancer multidisciplinary team meetings [Doctoral thesis, Imperial College London].” Spiral Repository, https://doi.org/10.25560/79603 by Soukup (2017).

### 3.3. Q3: Is there a difference in communication in the first half of the meeting vs. the second half?

The frequency and percentage change for each feature of communicative dysfluency between the first and the second halves of meetings are presented in Table 6. An overall increase in verbal fragmentations of 52% in the second half of the meeting can be seen, with some variation between teams. For example, the colorectal cancer MDT showed the highest percentage increase in incomplete sentences, while the breast and gynecologic cancer MDTs showed an increase in interruptions, chatter, and laughter. In contrast, the breast cancer MDT showed the least increase in vocalizations, the gynecologic cancer MDT in raised pitch, and the colorectal cancer MDT in pauses. Moreover, the colorectal MDT was the only participating team where both the pathologist and oncologist used a videoconferencing system and were not physically present at the meeting. Here, there were frequent connection and sound issues, and raised pitch may have been used for clarity, resulting in a similar number of counts between the first and the second halves of the meeting with a small percentage change (–0.9).

**Table 6:** Frequency and percentage increase in verbal fragmentation in the first vs. the second half of meetings across the participating cancer multidisciplinary teams (MDTs).

	**Multidisciplinary cancer team**											
	**Breast**			**Colorectal**			**Gynecologic**			**Full sample**		
**Verbal fragmentation**	**1st half** ***n***	**2nd half** ***n***	**% increase**	**1st half** ***n***	**2nd half** ***n***	**% increase**	**1st half** ***N***	**2nd half** ***n***	**% increase**	**1st half** ***N***	**2nd half** ***n***	**% increase**
Incomplete sentences	*22*	*74*	237	*10*	*22*	120	*18*	*22*	22	*50*	*118*	136
Pauses	76	152	100	90	109	21	80	92	15	246	353	44
Pitch peaks	268	506	87	212	210	–0.9	209	222	6	689	938	36
Repetition	12	20	67	16	20	25	15	20	33	43	60	40
Vocalization	37	43	16	42	58	38	27	41	52	106	142	34
Interruption	7	19	171	4	6	50	2	6	200	13	31	138
Chatter and laughter	5	21	320	0	0	–	0	11	1,000	5	32	540
Overall	427	835	96	374	425	14	351	414	18	1,152	1,674	45

Analysis was conducted on 24 case discussions. The average *duration of the meeting* was 60 min for the breast cancer team, 45 min for the colorectal cancer team, and 160 min for the gynecologic cancer team (Soukup, 2017). Reprinted with permission from Soukup (2017) “Socio-cognitive factors that affect decision-making in cancer multidisciplinary team meetings [Doctoral thesis, Imperial College London].” Spiral Repository, https://doi.org/10.25560/79603 by Soukup (2017).

For the three teams combined, the chatter and laughter, interruptions, and incomplete sentences showed the greatest increase. Approximately a 1-fold increase was evident in incomplete sentences, a 1.5-fold increase in interruptions, and nearly a 4-fold increase in chatter and laughter in the second half of the meeting. This was closely followed by pauses, repetitions, vocalizations, and pitch peaks with the smallest increases.

## 4. Discussion

Guided by some of the analytical principles of linguistics and CA, our study explored the communication patterns that underpin patient decision-making in cancer MDT meetings.

### 4.1. Q1: Is there an identifiable pattern of who leads or initiates conversation in the meeting?

We found that across teams, surgeons were the most frequent initiators and responders of interaction sequences, while CNSs and coordinators were the least frequent. Oncologists were also high-frequency contributors in breast MDT meetings, whereas, in colorectal and gynecologic meetings, communication was driven solely by surgeons. This finding is consistent with previous studies showing that surgeons, and to a lesser extent oncologists, are the most frequent contributors to case discussions in the meetings, while CNSs and coordinators do not contribute to the same extent (Lamb et al., 2011, 2013; Raine et al., 2014; Soukup et al., 2016a, 2021a; Soukup, 2017). However, while coordinators have an administrative role and their input into case discussions is not expected, the input of CNSs is required and is often critical to decisions around care planning. Moreover, in the breast and gynecologic team meetings, communication was driven by declarative statements, with statements/giving information appearing to be the most common way of initiating sequences of interaction by both initiators and responders. In the colorectal meetings, communication was more dominated by question–answer pairs. Here, the initiators would largely use an interrogative form of communication, and the responders a declarative one.

### 4.2. Q2: How responsive are team members to each other during case discussions?

We found that for every sequence of interactions initiated, a member received a response from the team. In breast cancer meetings, in particular, the responsiveness appeared to be even higher, with the initiator receiving an average of one and a half responses for each initiated sequence of interactions. This points to MDT meetings exhibiting high levels of interactivity, which is in line with previous findings in this setting (Soukup et al., 2021a,c).

### 4.3. Q3: Is there a difference in communication in the first half of the meeting vs. the second half?

A trend of increase in verbal fragmentations in the second half of meetings across participating teams was observed, with only slight variations. For instance, pitch peaks in the colorectal team meeting did not differ between the two time points, which could be due to the way these meetings are set up, with the oncologist and pathologist having to dial into the meeting, with Internet/sound issues a common occurrence. In the combined sample, however, the chatter and laughter, in addition to interruptions and incomplete sentences, seemed to be the most common across teams. These were closely followed by pauses, repetitions, and vocalizations, indicating less focused discussion in the second half of the meetings, pointing to a possible link to increased cognitive load and fatigue (Adda-Decker et al., 2008; Heldner and Edlund, 2010; Nicholson et al., 2010; Womack et al., 2012), and time-on-task effects on communication and decision-making in MDT meetings (Lamb et al., 2013; Soukup et al., 2019a,b, 2020a). It is possible that such effects also impacted the quality of decisions made—while the current study did not investigate this aspect, this is something that future research should further unpack to ascertain the correlation between the quality of the decision-making process and decisions made in relation to these effects. Further research should also examine the verbal fragmentations in more detail and their impact on team communication and decision-making in a larger sample and across more teams to understand the extent to which some of the patterns identified in our study apply to them.

### 4.4. Implications and further research

#### 4.4.1. Cognitive fatigue and quality of communication and decision-making in MDT meetings

The possible link between higher frequencies of verbal fragmentations, and increased cognitive load and fatigue, may also be a factor shaping team interaction (Adda-Decker et al., 2008; Heldner and Edlund, 2010; Nicholson et al., 2010; Womack et al., 2012). Verbal fragmentation can impact the listener's understanding of what the speaker wants to communicate to the group (Bailey and Ferreira, 2003; Barr and Seyfiddinipur, 2010; Womack et al., 2012; Soukup, 2017). Information that is not clearly communicated/understood can have an impact on clinical decision-making (Leonard et al., 2004; Soukup et al., 2016a,b, 2020a; Soukup, 2017). To optimize safety and quality, it is therefore important to maintain an acceptable level of cognitive load in MDTs during their weekly meetings by adapting appropriate cognitive strategies (Soukup, 2017; Soukup et al., 2019b). For instance, a short break in the middle of the meeting (Soukup, 2017; Soukup et al., 2019b), streamlining the workload according to clinical complexity using validated tools and clinical protocols (NHS England NHS Improvement, 2020; Soukup et al., 2020c,d), and a trained, non-contributing chair to facilitate communication and helping the team stay on task by minimizing the chatter, interruptions, and incomplete sentences (Soukup, 2017; Soukup et al., 2019b).

#### 4.4.2. Task complexity and cognitive load in MDT meetings

Another related point to consider is that in task-orientated interactions (such as those occurring in the context of MDT meetings where the task is to formulate treatment recommendations), speakers and listeners spend considerable time on task-relevant activities (e.g., going through patients' paper notes in the meeting, looking for radiology/pathology slides to upload, and taking notes/populating patient proformas) than on other speakers/team members. This is in contrast to spontaneous non-task-oriented interactions, where the focus is more on other speakers; hence, gaze/token responses are common (Nicholson et al., 2010). It is arguable, therefore, that fragmentations and disfluencies during case discussions may occur due to task or case complexity (Bard et al., 2001; Nicholson et al., 2010; Soukup, 2017). However, at the time of this study, psychometrically sound tools for gauging case complexity in MDT meetings were lacking. Instead, we matched the cases on, for example, malignancy and duration of case discussion. However, the cases will differ on finer clinical aspects and complexity (Soukup, 2017; Soukup et al., 2020c). Further studies are, therefore, needed to begin to build the knowledge base on this issue and to create a cohort of case discussions that are closely matched on clinical complexity—something that can now be measured, for example, using the MeDiC tool (Soukup et al., 2020c). For instance, some of the questions that future studies could address are:—how do disfluencies differ in complex vs. simpler cases?—how do these change in the second half of the meetings? This would certainly begin to shed light on the relationships between verbal disfluencies and cognitive load/fatigue, and how they are elicited in the context of cancer MDT meetings (Soukup, 2017).

#### 4.4.3. Role and contributions of cancer nurse specialists in MDT decision-making

Hierarchy may shape interaction in these meetings in ways that indicate how participants orient to status, role, and responsibility. This needs to be evaluated further, for example through a direct assessment of levels of real and perceived hierarchy in cancer MDTs and how this may correlate with patterns of team communication as assessed in the present study. Further CA research may help to clarify this, by shedding light on how the hierarchy of clinical expertise may shape the form and content of interactions in MDT meetings. CNSs, for example, occupy a lower professional status within this hierarchy, which appears to reflect their level of direct contribution. However, as discussed, their role is often critical, and one example from our data shows a direct contribution from a CNS that resulted in a change in the original decision (e.g., from discharging the patient to a clinical review). Communication in MDT meetings is influenced by many factors, including hierarchy, status, and power relationships. Our data appear to indicate that the hierarchy of expertise within the MDT does not determine action, but may systematically shape how communication between team members is conducted. Further analysis of this would shed light on the relationship between hierarchy and perceptions of role and responsibility.

#### 4.4.4. Integration of patient perspectives into MDT decision-making

Further understanding is needed of how patients' perspectives are incorporated into MDT decision-making across different teams and how this could be optimized (during and post-MDT meetings; Soukup et al., 2021b). This is particularly important in light of the current study, and previous research, demonstrating their underrepresentation (Lamb et al., 2011, 2013; Raine et al., 2014; Stairmands et al., 2015; Soukup et al., 2016a,b, 2020a,b, 2021c). It is understood that patients are experts in their health and lived experience and that they should be considered equal partners in clinical decision-making (Department of Health, 2004; Landmark et al., 2015; Soukup, 2017). This is reflected in the recommendations for MDTs suggesting a patient-centered approach (Department of Health, 2004), so that their views are included in the MDT discussion as part of the minimum information required about the patient (National Cancer Action Team, 2010), and shared decision-making as a healthcare norm (Department of Health, 2012).

### 4.5. Limitations and generalizability

Our study has limitations, some of which have been reported previously (Soukup, 2017; e.g., Soukup et al., 2019a, 2020a,c, 2021a). The first is the Hawthorne effect. We minimized its effect by (a) using a long-term approach to filming, (b) excluding the first two meetings from the analysis, and (c) filming discreetly (Soukup, 2017; Soukup et al., 2021a). Second, there were instances of inaudible speech in the meetings of all participating teams. This is a natural limitation of such complex multiparty interactions, where people do not speak in neatly organized rounds (Soukup, 2017; Soukup et al., 2021a).

However, by using real-time, unstructured observations of cancer teams, we were able to capture the flow of behavior in its setting, thus achieving greater ecological validity, while generating new avenues of inquiry that may provide new insights for improving MDT meetings and a better understanding of teams in general (Soukup, 2017). Our study also shows that a hybrid approach, encompassing qualitative data and quantitative frequency counts, is a feasible method for studying MDT communication and complex team dynamics. Future studies could apply our method to a larger sample to help build knowledge and generalizability in the context of cancer MDT meetings, as well as across other chronic conditions that use MDT meetings (Soukup, 2017).

Finally, we did not examine the effect of individual team members in the meetings. We acknowledge that although this is important to explore, it also carries a certain risk in potentially and unintentionally creating a culture of blame. We have, therefore, focused on disciplinary/professional groups, which is helpful when studying relatively small teams, such as the MDTs, because it ensures team safety by minimizing the risk of defensive routines and blaming a particular member for performance difficulties, which could distract from addressing the issues constructively (West, 2012; Soukup et al., 2019a). Similarly, and consequently, we did not collect information on the individual members' qualifications or years of experience in their current role, except that the members' studied as part of the analysis presented in the current study were at the consultant level, as they were more formally considered to be the core members who actively participated in and led the discussion. We know, however, that there are professional hierarchies and that more junior doctors may be present at MDT meetings but are not empowered to speak (West, 2012). Future studies should explore this aspect in more detail, with MDT research incorporating the hierarchies into the study design, which would allow for a more granular assessment of how different hierarchical positions impact team decision-making. In a similar vein, understanding the role of preparation time for MDT meetings and how this might impact the level of verbal contribution of team members to the discussion should also be further investigated, as this cannot be concluded from the current study and should be taken into consideration when interpreting the participation of different professional groups.

## 5. Conclusion

Factors such as (a) team cognitive load and fatigue, and (b) CNSs' input should be considered when planning MDT meetings because of their potential impact on the quality of team communication and decision-making. Our methodological approach could be further applied to other healthcare teams to build a knowledge base on team communication in this and other settings, and to provide guidance to teams to optimize teamwork.

## Data availability statement

The datasets presented in this study can be found in online repositories. The names of the repository/repositories and accession number(s) can be found below: https://zenodo.org/record/582272#.XntHvoj7Q2w.

## Ethics statement

The studies involving human participants were reviewed and approved by Northwest London Research Ethics Committee (JRCO REF. 157441). The patients/participants provided their written informed consent to participate in this study.

## Author contributions

TS and GM have made substantial contributions to the conception and design of the study. All authors have made a substantial contribution to data acquisition, analysis, and interpretation of data, have been involved in drafting the manuscript and revising it critically for important intellectual content, have given final approval of the version to be published, and have agreed to take responsibility for all aspects of the article to ensure that questions relating to the accuracy or integrity of any part of the article are appropriately investigated and resolved.
